# Demonstrating voxel-by-voxel (V × V) single-point calibration in liver tissue by IR-MALDESI quantitative MSI

**DOI:** 10.1007/s00216-025-06138-x

**Published:** 2025-09-29

**Authors:** Emily R. Bruce, Russell R. Kibbe, Logan J. Opperman, David C. Muddiman

**Affiliations:** 1 Biological Imaging Laboratory for Disease and Exposure Research, Department of Chemistry, North Carolina State University, Raleigh, NC 27695, USA; 2 Department of Statistics, North Carolina State University, Raleigh, NC 27695, USA

**Keywords:** IR-MALDESI, Quantitative mass spectrometry imaging, Single-point calibration, Quantitative sampling

## Abstract

Quantitative mass spectrometry imaging (qMSI) provides information regarding the colocalization, relative abundance, and concentration of a target analyte in a tissue without homogenization. Ionization sources, including IR-MALDESI, commonly utilize an on-tissue spatial calibration curve approach; however, this approach has several limitations including tedious sample preparation, and this approach does not account for local matrix effects. To compensate for these two limitations, we developed voxel-by-voxel (V × V) quantification to provide an internal standard calibration point for every voxel which requires a simple sample preparation and accounts for local matrix effects. In this work, we evaluate the performance of V × V quantification against the spatial calibration curve to assess the quantitative capacity of this newly developed method. Quantification of glutathione (GSH) on a per-voxel basis involves homogenously spraying a known amount of stable isotope-labeled glutathione (SIL-GSH) on a microscope slide. Next, we mount liver sections on top of the coated slides and image them using IR-MALDESI MSI. Statistical analysis demonstrated high precision for V × V quantification over a wide concentration range; however, the method’s accuracy is currently limited due to the sprayer’s configuration. Results support the feasibility of V × V quantification as evidenced by concentration heatmaps. Additionally, V × V quantification allows for parallel reaction monitoring (PRM) imaging which provides high specificity. Combined with relativity, straightforward sample preparation, and promising initial statistics, the V × V method offers significant advantages over spatial calibration curves.

## Introduction

Mass spectrometry imaging (MSI) is a powerful tool that enables the visualization of morphological features in tissues while simultaneously mapping the spatial distribution of hundreds to thousands of analytes without the need for labeling [1]. MSI is rapidly evolving and contributes significantly to a wide range of biological studies [2]. This process involves sampling biological tissues and analyzing them using mass spectrometry while preserving spatial context, resulting in ion heatmaps that depict the localization of specific analytes [1].

In the mass spectrometry field, liquid chromatography-tandem mass spectrometry (LC–MS/MS) serves as the gold standard for quantifying a target molecule from diverse molecular classes; however, the required tissue homogenization leads to a loss of spatial information [1]. Given MSI’s ability to identify, localize, and quantify specific biomarkers within tissue, there is a growing interest in developing and optimizing techniques for absolute quantification [1]. Quantitative mass spectrometry imaging (qMSI) plays an increasingly important role in areas such as metabolomics [3] and drug discovery [4].

Several techniques exist for performing qMSI [5], with the on-tissue spatial calibration curve being the most widely used. This method involves preparing a serial dilution of a stable isotope labeled (SIL) version of a target analyte and spotting the dilution series on top of the tissue of interest [5]. Although the spatial curve accounts for global matrix effects when applied to tissue [5], it introduces several limitations particularly in heterogeneous tissues. For example, spotting a spatial calibration curve can be especially challenging when working with small tissues since it is best practice to spot in the region of interest being quantified, as this helps account for density, cell type, and structural heterogeneity. In addition to this challenge, tissue heterogeneity also brings rise to differing ionization efficiencies where one spatial calibration curve spotted for a specific region of interest may not accurately reflect the analyte’s concentration in other regions of the tissue. To overcome these challenges, researchers have proposed alternative approaches such as multi-labeled per-pixel quantification. This approach involves homogenously spraying three different labeled standards at differing concentrations, creating a 3-point calibration curve at every voxel [6]. In addition to this approach, researchers have also proposed utilizing a tissue extinction coefficient (TEC) as a normalization factor since a major limitation to qMSI is ion suppression as well as the availability of isotopically pure stable isotope labeled standards [7]. This approach normalizes the mean tissue abundance by dividing the average abundance of the tissue region by that of the slide region and then applies this normalized value to the calibration equation to determine the analyte concentration [7]. These methods have been shown to be advantageous to heterogenous tissues but can become very costly and time-consuming when quantifying more than one tissue. As a result, a simplified qMSI technique is needed with comparable accuracy as well as reduced cost and experimental complexity.

Voxel-by-voxel (V × V) quantification is an alternative qMSI approach and enables single-point calibration at every voxel within a tissue of interest. This method consists of two simple steps: homogenously spraying a known amount of SIL standard on a clean microscope slide followed by thaw mounting the tissue of interest on top of the sprayed slide. Next, since we achieve quantitative sampling of the tissue at each voxel [8], the abundance ratio between the target analyte and the SIL standard is all that is required for the calculation to determine the concentration of the endogenous analyte on a per voxel basis. Compared to the spatial calibration curve, V × V offers several advantages including a straightforward sample preparation, the ability to account for both global and local matrix effects, reduced complexity in data analysis due to fewer sources of error (e.g., selecting the ROI for each calibration point), and compatibility with PRM-qMSI which enhances molecular specificity.

Multiple ionization sources have demonstrated the feasibility of absolute qMSI. Matrix-assisted laser desorption/ionization MSI (MALDI-MSI) remains the most commonly used ionization source for qMSI, but users must account for signal variability caused by heterogenous matrix crystallization [9]. Conversely, ionization sources such as desorption electrospray ionization (DESI) demonstrated the feasibility of absolute qMSI using a spatial calibration curve without requiring matrices [10]. Similarly, infrared matrix-assisted laser desorption electrospray ionization (IR-MALDESI), a hybrid ionization source that combines the benefits of both MALDI and electrospray ionization (ESI), demonstrated absolute qMSI by facilitating the formation of a thin ice matrix on top of the sample of interest [11–13].

Previous IR-MALDESI qMSI studies quantified the total concentration of glutathione in healthy and cancerous hen ovarian tissue, as well as mouse liver, using a spatial calibration curve [11, 12]. To date, this approach remains the only quantitative approach demonstrated by IR-MALDESI. More recently, IR-MALDESI confirmed quantitative sampling by achieving full tissue ablation with each laser ablation event, enabling the application of the normalization and internal standards under the tissue of interest [8]. Building on these advancements and previously optimized concentrations of glutathione [11], the present study takes a systematic approach to evaluate the performance of this newly developed quantification approach, V × V quantification, against the widely used spatial calibration curve to determine the quantitative potential of this alternative approach.

## Methods

### Materials

LC–MS grade water (H_2_O), methanol (MeOH), and formic acid (FA) were purchased from Fisher Scientific (Nazareth, PA) for use in electrospray solvent. Stable isotope-labeled glutathione (^13^C_2_^15^N – SIL-GSH, Item #: CNLM-6245-HP-10, Net Purity: 90%) was purchased from Cambridge Isotope Laboratories (Tewksbury, MA). Native glutathione (NAT-GSH) was purchased from Millipore Sigma (Burlington, MA), and homoglutathione (hGSH) was purchased from Bachem (Torrance, CA).

### Sample preparation

**Fig. 1:**
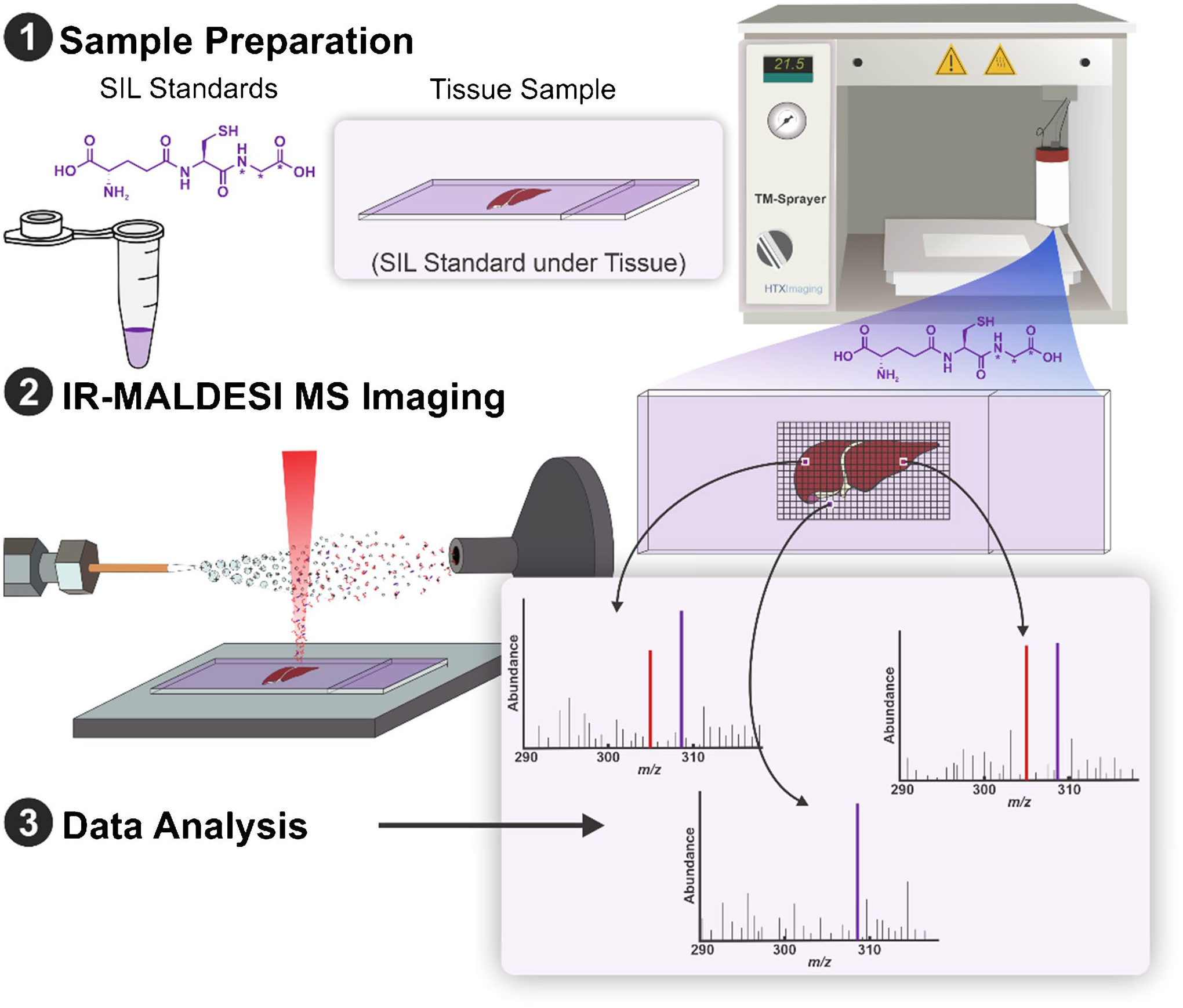
Schematic of experimental design for V × V quantification by IR-MALDESI qMSI. A TM sprayer homogenously sprayed stable isotope-labeled glutathione on a microscope slide to serve as an internal standard. The protocol then involved sectioning a mouse liver and thaw-mounting it onto the sprayed slide. The prepared slides underwent immediate analysis by IR-MALDESI. Data analysis calculated the abundance ratio of the target and internal standard analyte and multiplied by the known amount of internal standard at each voxel to yield the concentration of the target analyte at each voxel

Figure 1 shows the schematic of the experimental design for V × V quantification by IR-MALDESI qMSI.

#### Spatial calibration curve—glutathione (GSH) quantification

The Ghashghaei lab in the College of Veterinary Medicine at North Carolina State University (NCSU) provided a healthy, wild-type, fresh-frozen mouse liver. NC State University Institutional Animal Care and Use Committee (IACUC, #19–811-B) approved all animal experiments. Similar to previous experiments [11], we utilized a Leica CM1950 cryostat (Buffalo Grove, IL) at −15 °C to serial section the fresh-frozen liver at 7 μm [14]. Before mounting the liver section to a glass slide, a pneumatic sprayer (TM Sprayer, HTX Technologies, Carrboro, NC) homogenously sprayed 1.0 mg mL^−1^ homoglutathione in 50% methanol onto microscope slides to act as a normalization standard. Next, we carefully thaw-mounted the sectioned liver onto the coated slides and prepared a serial dilution series of SIL-GSH at concentrations of 0, 0.0313, 0.0625, 0.125, 0.250, 0.500, and 1.00 mg mL^−1^ in 50:50 (v/v) methanol/water. Each solution from the dilution series was spotted on top of the liver section in discrete and non-overlapping locations (7 spots total) at a volume of 100 nL by using a Hamilton 7000.5 syringe (Hamilton Company, Reno, NV). IR-MALDESI immediately imaged the prepared samples.

#### Voxel-by-voxel (V × V) quantification—glutathione (GSH) quantification

V × V quantification utilized the same fresh frozen mouse liver obtained for the spatial calibration curve. Before sectioning and thaw mounting the liver on microscope slides, we homogenously sprayed 1.0 mg mL^−1^ SIL-GSH onto microscope slides to act as an internal standard at every voxel within the mouse liver section. All experiments used the following spraying conditions: 0.01 mL min^−1^ flow rate, 45 °C, 500 mm min^−1^, 3-mm track spacing, and four passes. From there, we sectioned the mouse liver at 7 μm [14], thaw-mounted the liver to the sprayed slide, and immediately analyzed by IR-MALDESI. This experiment investigated a total of two liver replicates for each qMSI method. Due to the architecture of the TM sprayer, the total volume sprayed is unknown, systematically inflating the endogenous concentration; the TM sprayer exhausts an unknown volume of solution depending on the percent organic.

#### Voxel-by-voxel (V × V) quantification—statistical analysis

To assess the precision of V × V quantification, we performed statistical analyses by preparing a dilution series of 2:1 solution of NAT-GSH and SIL-GSH where the total analyte concentrations in solution were 0, 0.0313, 0.0625, 0.125, 0.250, 0.500, and 1.00 mg mL^−1^ in 50:50 (v/v) methanol/water. From there, the pneumatic sprayer homogeneously sprayed each respective solution on a clean microscope slide. IR-MALDESI measured the prepared slides.

Additionally, to validate the robustness of this method, we performed a similar experiment and prepared a serial dilution of SIL-GSH at total concentrations of 0, 0.0313, 0.0625, 0.125, 0.250, 0.500, and 1.00 mg mL^−1^ in 50:50 (v/v) methanol/water. The TM sprayer homogenously sprayed each concentration on a respective clean glass slide. From there, we sectioned fresh frozen mouse liver at 7 μm ^14^ for each sprayed slide and carefully thaw-mounted them on top of the sprayed slide. Likewise, IR-MALDESI imaged the prepared samples.

### IR-MALDESI MSI

The Next Generation IR-MALDESI source coupled to an Orbitrap Exploris 240 Mass Spectrometer (Thermo Fisher Scientific, Bremen, Germany) analyzed the tissues [15]. We described the details of the NextGen IR-MALDESI source elsewhere [15, 16]. In short, we place prepared samples on an XYZ-controlled Peltier stage, which is housed in an acrylic enclosure. Next, nitrogen gas purges the enclosure to reduce the relative humidity (RH), and the Peltier stage cools down to −8 °C. After holding the temperature constant for several minutes to reach thermal equilibrium, an ice matrix forms on top of the samples as the enclosure opens and is exposed to ambient humidity. To prevent continual ice growth, we close the enclosure and purge with nitrogen, until the RH decreases back to ~ 10%. This source utilizes a mid-IR laser with a wavelength of 2.97 μm which is the wavelength required to resonantly stretch the O–H bonds of the endogenous water molecules within the sample as well as the exogenous water molecules from the ice matrix. When the mid-IR laser fires, neutral molecules desorb, and an orthogonal electrospray ionization intercepts and ionizes the neutrals in an ESI-like manner.

In qMSI experiments, the optical train includes a diffractive optical element (DOE) and a 25-mm FL aspheric lens to accomplish top-hat imaging, resulting in square ablation spots [11, 17]. This optical train resulted in spot sizes approximately 140 × 140 μm. All experiments utilized negative mode with the electrospray composed of 80% methanol in water with 0.1% formic acid [11, 18]. The solution was infused at 2.0 μL/min and stabilized at approximately 3400 V. Additionally, the instrument operated at a resolving power of 240,000_FWHM_ at *m/z* 200. For all experiments, the scans were acquired over a *m/z* range of 200 to 400, and the data was collected in centroid mode. Automatic gain control was disabled and used a 15 ms ion injection time.

### Data analysis

ProteoWizard’s MS Convert [19] converted the raw data files into.mzML files. From there, imzML Converter [20] converted the files into imzML format. MSiReader Pro v3.07 (MSI Software Solutions, LLC, Raleigh, NC) opened and analyzed the imzmL files [21, 22]. QC check ensured high mass measurement accuracy was achieved, and the produced ion images had a ± 2.5 ppm *m/z* tolerance. Additionally, for both approaches, the net purity of SIL-GSH (90%) was accounted for by adjusting all input concentrations downwards by 10% during data analysis.

#### Spatial calibration curve

The absolute quantification function in MSiReader calculated the average absolute concentration of endogenous glutathione in the mouse liver. To increase the sample size (*n* = 30) for the replicate tissue, we employed square bioinformatic regions of interest (ROIs) within each replicate which allowed the sample size to increase to 30 for each replicate. From the thirty ROIs, each containing 20 scans, the average normalized abundance of endogenous GSH in each ROI was utilized to calculate the average concentration of endogenous GSH in that specific area. With these average concentrations, we performed a subsequent statistical comparison between three normalization conditions. Details of this process are described in previous work [11].

#### Voxel-by-voxel (V × V) quantification tool

**Fig. 2:**
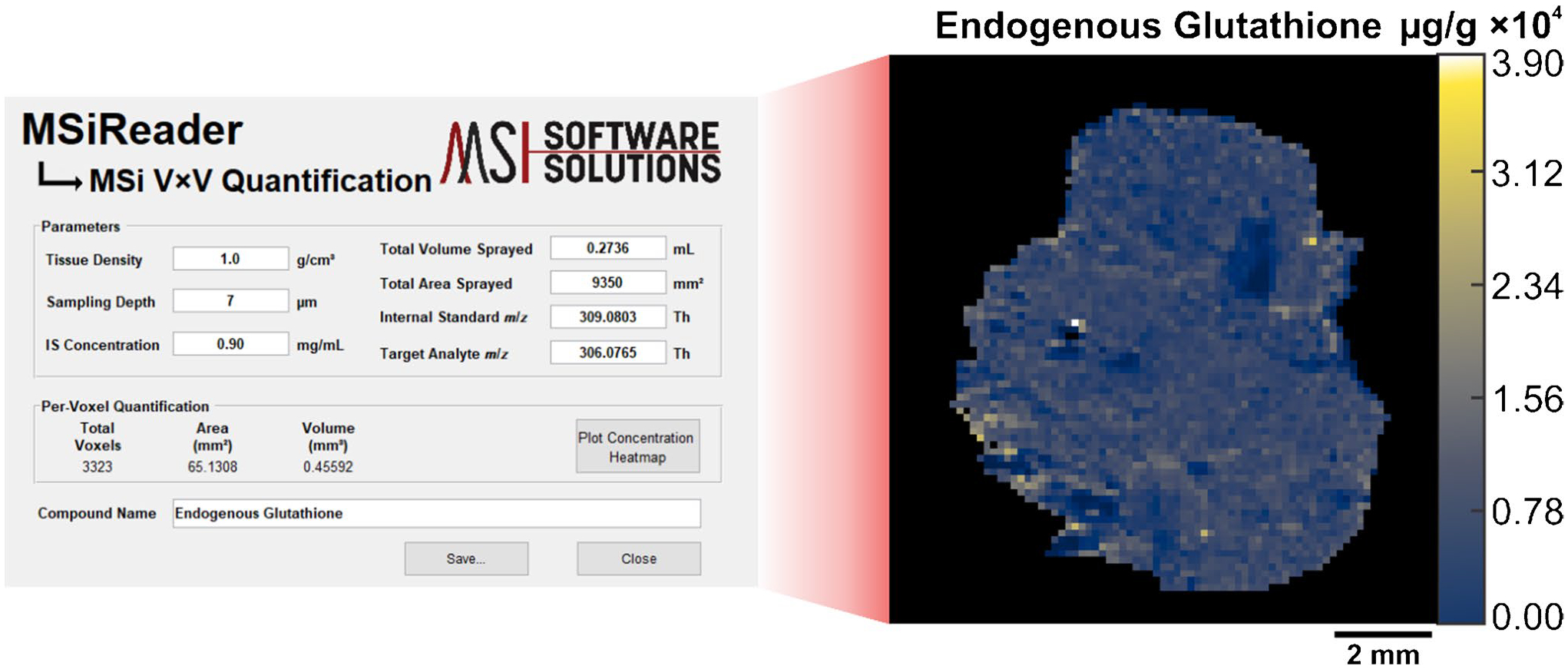
The graphical user interface in MSiReader for V × V quantification along with an outputted concentration heatmap of glutathione. The outputted concentration heatmap depicts the absolute concentration of endogenous glutathione on a μg_endogenous_/g_tissue_ scale

To output the concentration of endogenous GSH on a per-voxel basis, a newly developed tool in MSiReader outputs the concentration of endogenous GSH on a per-voxel basis and generates a corresponding concentration heatmap. This function, called MSi V × V quantification, determines the endogenous concentration of our target analyte at every voxel by taking the ratio of ion abundances of endogenous GSH and SIL-GSH at a specific scan and multiplying that value by the known amount of SIL-GSH at every voxel within the tissue. To generate a concentration heatmap of the target analyte, the method requires the following parameters: tissue density (g cm^−3^), sampling depth (μm), internal standard concentration (mg mL^−1^), total volume sprayed (mL), total area sprayed (mm^2^), and the *m/z* for the target and internal standard analyte. From there, the software generates a concentration heatmap of endogenous GSH with units of μg_endogenous_/g_tissue_. Additionally, this tool allows users to select any specific voxel within the tissue ROI, and the concentration at the respective scan is displayed. Figure 2 shows the schematic of the graphical user interface for MSi V × V quantification as well as the generated concentration heatmap.

## Results and discussion

### Quantitative results improve with a normalization standard

A spatial calibration curve can lead to several limitations including ion suppression and variation in tissue morphology, both of which can contribute to an increase in voxel-by-voxel variability [23]. Researchers proposed several methods to correct for these limitations including TIC normalization and employing a normalization standard. Although TIC normalization is widely used, incorporating a normalization standard in qMSI experiments is gaining popularity in the MSI community.

IR-MALDESI demonstrated success in applying a normalization standard underneath the tissue due to IR-MALDESI’s ability to achieve quantitative sampling [8]. When deciding on the ideal normalization standard for a target analyte, the normalization standard must remain exogenous, structurally similar, and occupy a different *m/z* space than the analyte of interest [13]. In this experiment, homoglutathione (hGSH) served as the normalization standard to correct voxel-by-voxel variability and improve quantitative results.

**Fig. 3:**
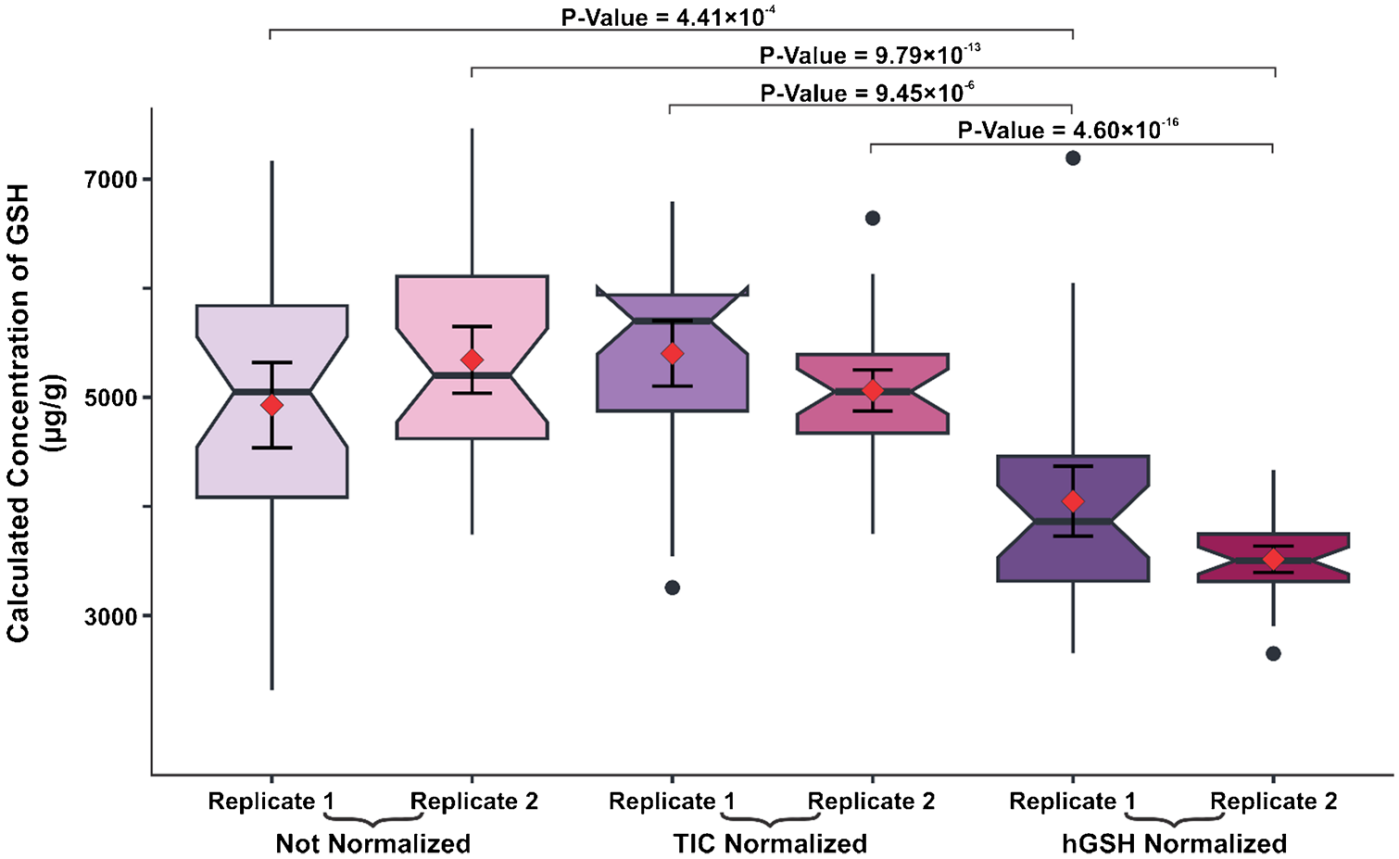
Notch-boxplots represent the calculated concentration of GSH for three different normalization conditions. The red diamonds represent the average concentrations, the error bars within the boxplots represent the 95% confidence intervals of the mean, and notches represent the 95% confidence intervals of the median. A two-sample *t*-test on all conditions indicated hGSH normalized data was significantly different than the unnormalized and TIC normalized data

In this work, a pneumatic sprayer sprayed 1.00 mg mL^−1^ hGSH onto a glass slide [12]. To assess whether the application of a normalization standard resulted in any statistically significant differences, we utilized notch box plots to illustrate each liver replicate for three conditions: unnormalized data, TIC normalized data, and data normalized to hGSH (Fig. 3).

Comparing the unnormalized and hGSH normalized data revealed a significant difference, as indicated by the lack of overlapping 95% confidence intervals of the mean and median. To further support this claim, we performed a two-sample *t*-test which yielded *p*-values of 4.41 × 10^−4^ and 9.79 × 10^−13^ for replicates 1 and 2, respectively. Likewise, the comparison of TIC and hGSH normalization showed statistically significant differences where one replicate provided the *p*-value of 4.60 × 10^−16^ and no observed overlapping of the 95% confidence intervals of the mean or median. Furthermore, the data normalized to homoglutathione illustrated a clear decrease in voxel-by-voxel variability and yielded average concentrations closer to reported literature values [24] emphasizing the importance of utilizing a normalization standard to improve the accuracy of the results.

### Quantification of glutathione in mouse liver

**Fig. 4:**
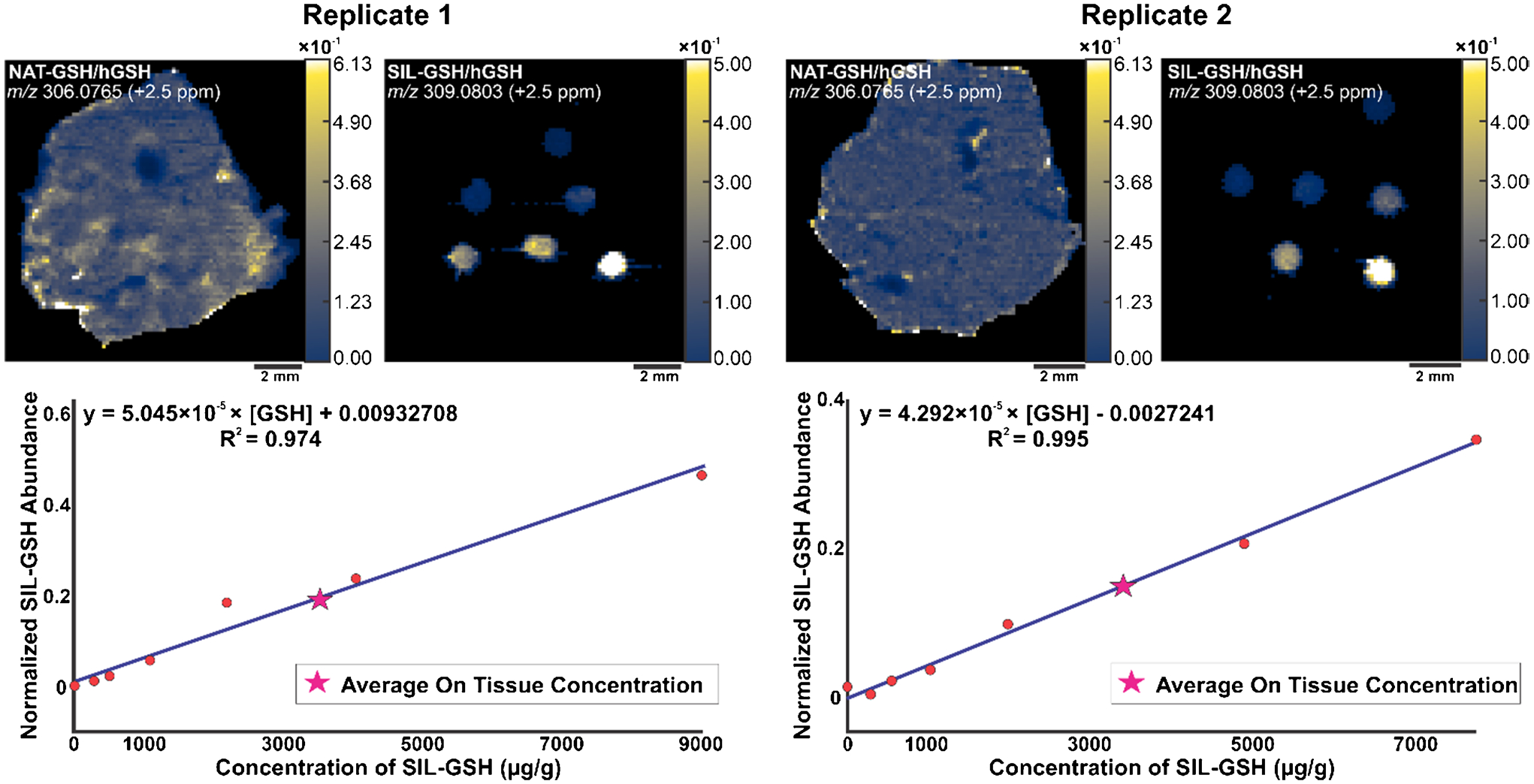
Ion heatmaps of NAT-GSH and SIL-GSH normalized to hGSH for both replicates in mouse liver (top). The spots from the SIL-GSH constructed the calibration curves (bottom) for the replicates. The pink star represents the average on tissue concentration of glutathione in μg/g

For the spatial calibration curve, we identically prepared two mouse liver replicates and analyzed them by IR-MALDESI. We prepared a serial dilution series of SIL-GSH and spotted the solutions on top of the liver tissue. These spots yielded a calibration curve from which the absolute concentration of endogenous GSH could be determined. The spotted dilution series on top of the tissues accounts for the global matrix effects due to the labeled standard behaving in the same manner when desorbed from the tissue of interest. The MSiQuantification tool, in MSiReader Pro v3.07, generated calibration curves for each liver replicate after normalizing the abundances of both endogenous GSH and SIL-GSH to hGSH (Fig. 4).

The *x*-axis of these calibration curves represents the concentration of SIL-GSH in units of μg of standard per gram of tissue while the *y*-axis represents the normalized abundance of the SIL-GSH spots. Replicate 1 had an average endogenous glutathione concentration of 3485.96 μg/g, while replicate 2 averaged 3468.97 μg/g. A two-sample *t*-test performed on the replicates yielded a *p*-value of 0.604, indicating no significant differences between them.

**Fig. 5:**
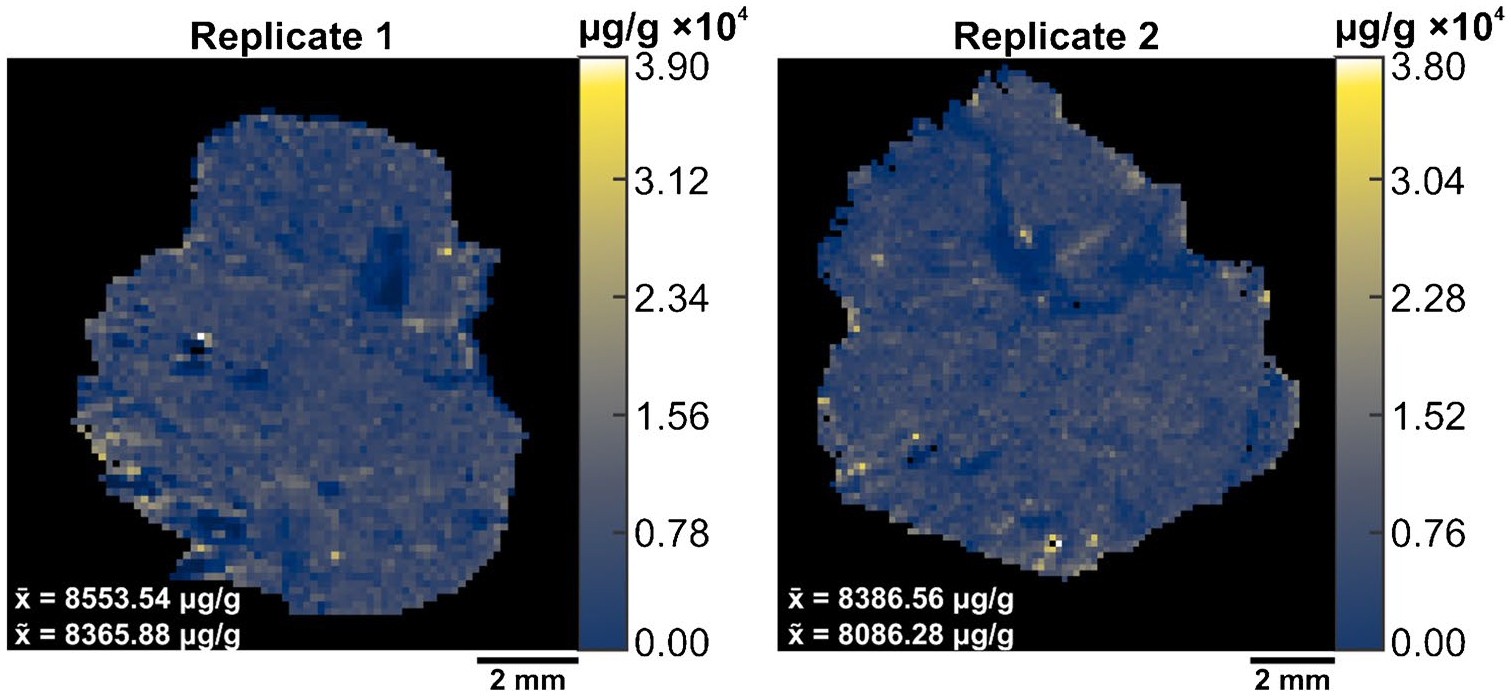
The outputted concentration heatmaps of endogenous glutathione (*m/z* 306.0765 ± 2.5 ppm) for liver replicates after V × V. The x-bar ($\bar{\text{x}}$) represents the average concentration of endogenous GSH while the x-tilde ($\tilde{\text{x}}$) represents the median concentration of GSH. Each voxel represents the concentration of GSH (μg/g) at a respective scan. The concentration scale is shown on the right side of each image. Tissue folding during mounting led to the exclusion of the edges in the liver tissue

To perform V × V quantification, we prepared two additional samples and imaged them by IR-MALDESI. A pneumatic sprayer homogenously sprayed 1.0 mg mL^−1^ of SIL-GSH onto microscope slides. Next, we sectioned and thaw-mounted the mouse liver on top of the sprayed slides. By spraying a known amount of SIL-GSH, the internal standard is present at every voxel which accounts for both global and local matrix effects. The MSi V × V quantification tool generated concentration heatmaps of endogenous GSH on a μg/g scale (Fig. 5).

The parameters inputted into this tool comprised of tissue density of 1.0 g cm^−3^, a sampling depth of 7 μm, an internal standard concentration of 0.90 mg mL^−1^, a total volume sprayed of 0.2736 mL, a total area sprayed of 9350 mm^2^, and finally, the internal standard *m/z* of 309.0803 alongside the target *m/z* of 306.0765. The concentration of GSH on a per-voxel basis averaged 8553.54 μg/g and 8386.56 μg/g for replicates 1 and 2, respectively. V × V quantification yielded higher concentrations than the calculated concentrations from the spatial calibration curve. This is attributed to the design of the sprayer used for this experiment which is known to not deposit the total volume dispensed through the nozzle depending on the solvent and temperature (Personal Communication, 2025, Alain Creissen, HTX Technologies, NC). Due to this issue, the total volume sprayed limits the accuracy of this method, and the direct comparison of quantitative approaches as well as literature values remains a challenge. The data presented in this paper assumes we are quantitatively spraying a known volume of solution onto microscope slides.

### Parallel reaction monitoring quantitative mass spectrometry imaging (PRM-qMSI)

Parallel reaction monitoring (PRM) is a widely utilized technique in the mass spectrometry field, known for enhancing signal-to-noise ratio, providing high specificity for tissue analysis, as well as offering the potential for faster acquisition times. More details of this technique are described elsewhere [25]. In short, during a PRM event, the quadrupoles select a specific *m/z* range, allowing ions within that range to exit the quadrupoles and enter the c-trap. These analyte ions proceed to the HCD cell, where they undergo collisions with nitrogen gas and are subsequently fragmented. The resulting fragments exit the HCD cell, reenter the c-trap, and then are injected into the Orbitrap for detection.

**Fig. 6:**
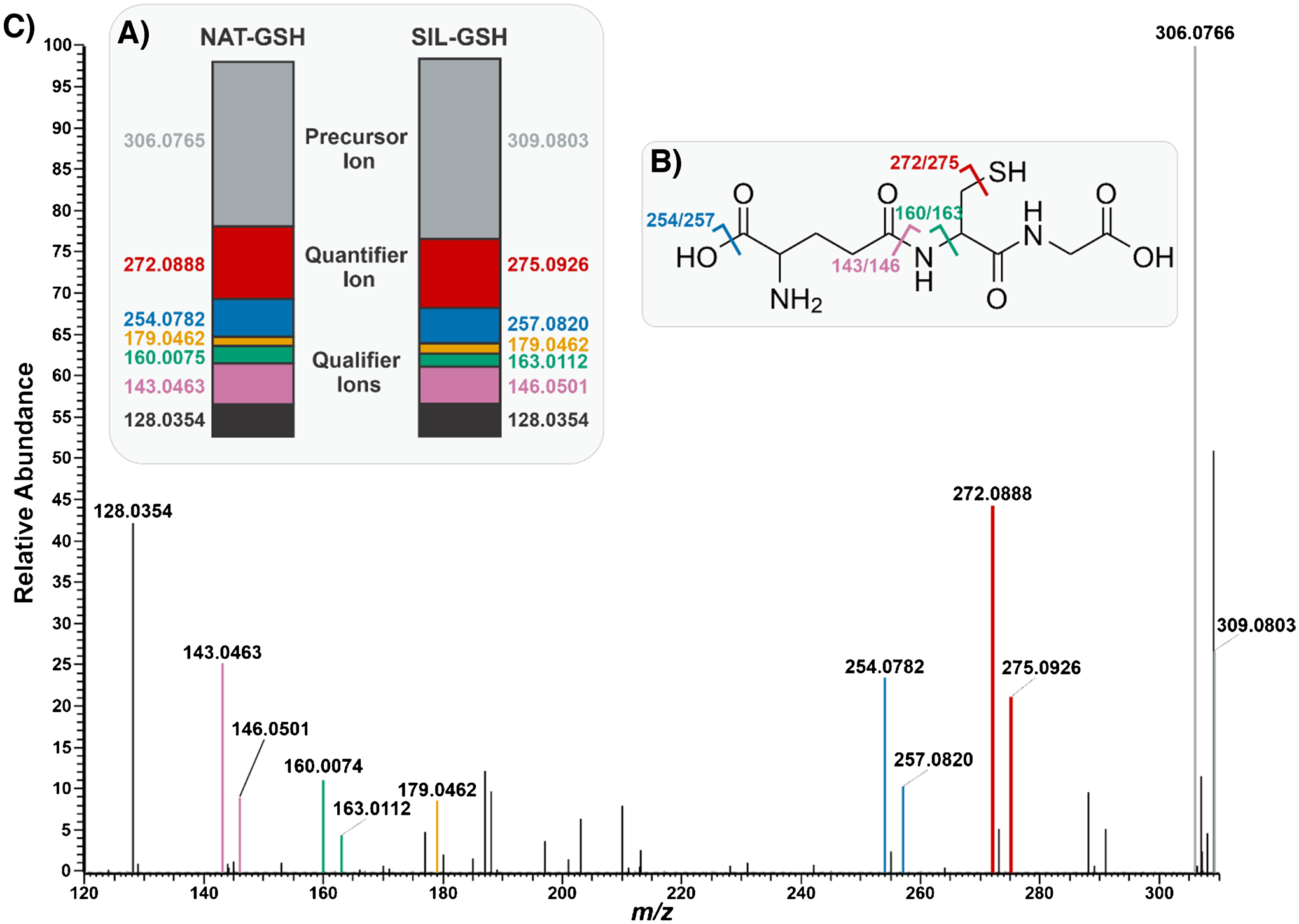
**A** Ion abundance ratios for NAT-GSH and SIL-GSH. The precursor ion is represented in gray, the quantifier peak chosen for the experiment is denoted in red, and the qualifier peaks are shown in other various colors. **B** Chemical structure of glutathione with annotated fragmentation sites corresponding to the observed doublet peaks and their respective *m/z* values. **C** PRM mass spectrum capturing the fragmentation pattern of NAT-GSH and SIL-GSH

Before attempting to apply PRM-qMSI to V × V quantification, we optimized the isolation width *m/z* and collision energy. PRM-qMSI requires the simultaneous passage of both the endogenous and SIL-GSH analytes. We set the precursor *m/z* to 307.8784, which is the average of 306.0765 (GSH) and 309.0803 (SIL-GSH), and selected the isolation width of 4 m*/z* which isolated both intact species as well as their M + 2 peaks. Next, we calculated the optimized collision energy of GSH. We determined the optimal collision energy (CE) by plotting the ratio of fragment ions to the sum of fragment ions plus precursor ions as a function of percent normalized collision energy (NCE). When the ratio is 0.5, we observed the optimal NCE at 20% for GSH. Once we optimized these parameters, we performed PRM on tissue to observe the fragmentation pattern of GSH. We observed the conserved ion abundance ratios for both endogenous GSH and SIL-GSH in PRM qMSI, confirming the identification of glutathione and implying little to no interferences (Fig. 6A). Additionally, we observed the fragmentation pattern of GSH where six clear fragments of GSH were identified. Out of the six fragments, we selected one as the quantifier peak while the others were considered qualifier peaks (Fig. 6B). The endogenous GSH quantifier peak appears at 272.0888 m*/z* while the SIL-GSH quantifier peak appears at 275.0926 m*/z*. This fragment forms as a result of H_2_S loss from the intact species.

Once we defined the quantifier peak for endogenous and SIL-GSH, we performed V × V quantification to determine the feasibility of PRM-qMSI. By using the MSi V × V Quantification tool, in MSiReader Pro v3.07, we were able to propagate a concentration heatmap of our intact species as well as our quantifier peak.

**Fig. 7:**
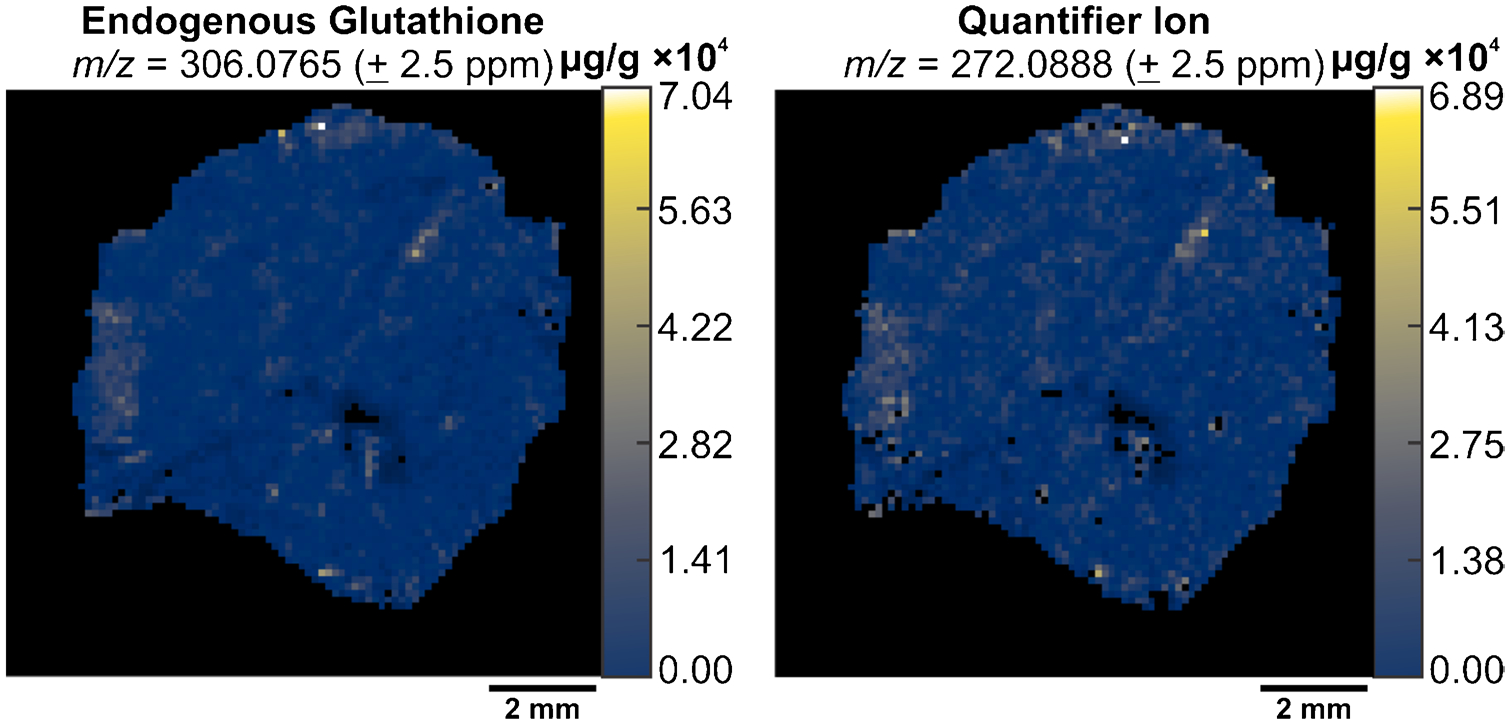
The generated concentration heatmaps of endogenous glutathione (m/z 306.0765 ± 2.5 ppm) (left) and the quantifier ion (m/z 272.0888 ± 2.5 ppm) (right). The concentration scale is shown on the right side of each image

The concentration heatmaps showed similarities evidenced by the homogenous distribution of endogenous GSH and similar absolute scalebars for both heatmaps. The use of the quantifier ion for V × V quantification improved confidence in analyte selectivity (Fig. 7).

### Statistical analysis

**Fig. 8:**
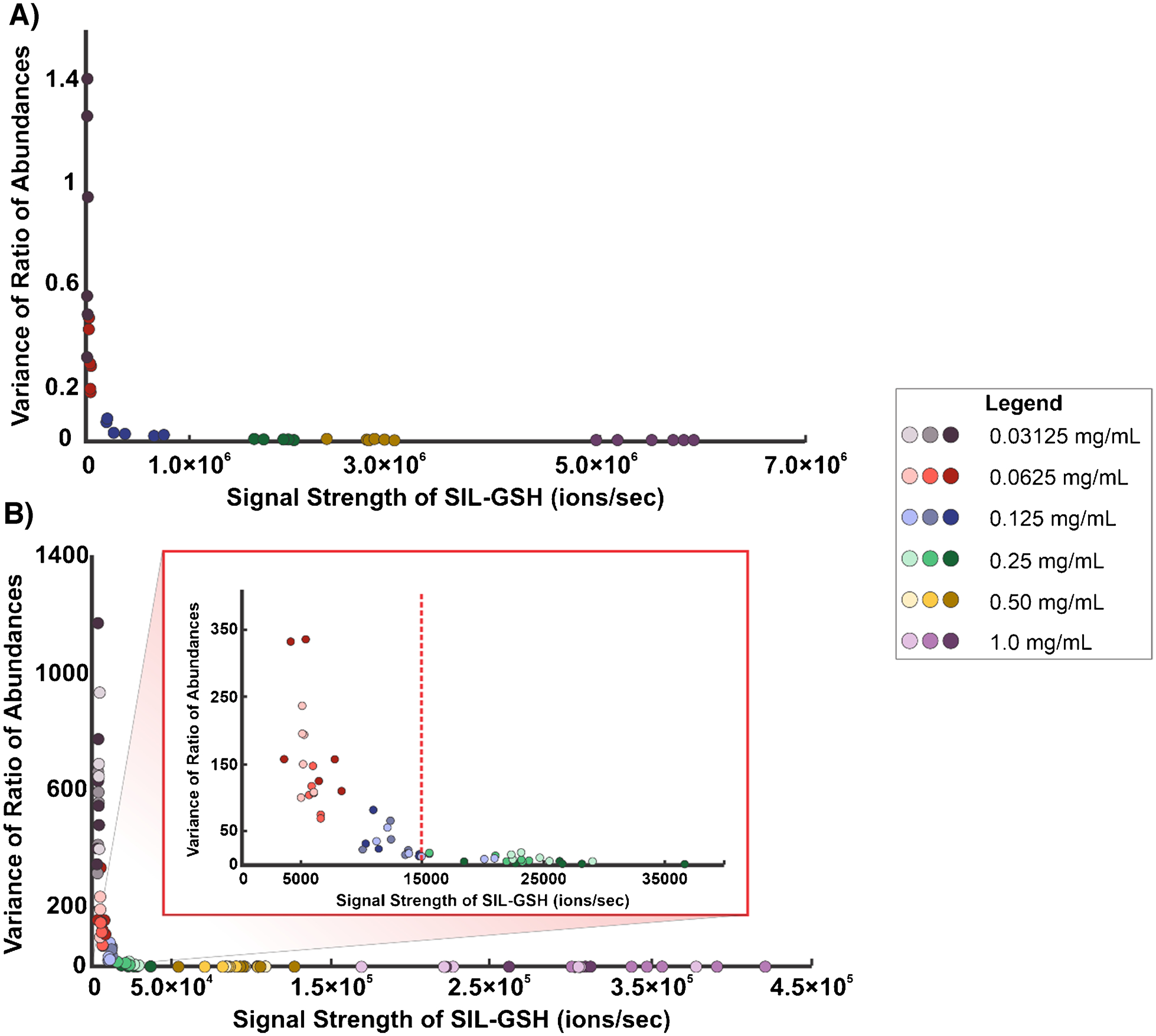
Scatterplots representing the variance of the ratio of abundances (NAT-GSH/SIL-GSH) plotted against the signal strength of SIL-GSH for **A** 2:1 dilution series of NAT-GSH:SIL-GSH and **B** the SIL-GSH dilution series with liver sections. In **B**, a zoomed-in region highlights the threshold, indicated by the red dotted line. The legend of the concentrations can be seen on the right side

To assess the variability of V × V quantification, we performed an experiment that involved preparing a dilution series of a 2:1 solution of NAT-GSH and SIL-GSH. With these solutions, we utilized the TM sprayer to homogenously spray each solution on a respective microscope slide. Next, we immediately sampled a 2704 scan ROI for each sprayed slide by IR-MALDESI. The variability of this method was first assessed by employing six square bioinformatic ROIs for each concentration and exporting the abundances of NAT-GSH and SIL-GSH. For each bioinformatic ROI, we calculated the average abundance of SIL-GSH, the abundance ratio between the two analytes, and the statistical variance of those ratios. In Fig. 8A, the relationship between the variance of the ratio of abundances and abundance of SIL-GSH can be observed.

Additionally, we performed a second experiment that introduced biological variability which involved preparing a dilution series of SIL-GSH, spraying each solution on a microscope slide by a TM sprayer, and mounting a liver section on top of the sprayed slide. Next, we imaged the liver sections by IR-MALDESI. For each concentration of internal standard, we prepared three replicates for a total of 18 tissue sections. Bioinformatic ROIs were employed and yielded the average SIL-GSH ion abundance as well as the abundance ratio between NAT and SIL-GSH. With that data, we calculated the variance of the abundance ratios. To visualize the variance of the abundance ratios, we plotted the variance of the ratio between NAT and SIL-GSH against the average ion abundance (ions sec^−1^) of SIL-GSH for each bioinformatic ROI (Fig. 8B).

These plots show a clear relationship between signal strength and the variance in the ratio of abundances. When the signal strength of SIL-GSH is low, the variance is large; however, it levels off once a certain threshold is exceeded. In Fig. 8B, the red dotted line indicates the signal threshold for precise and reliable data, set at 1.50 × 10^4^ ions per second. Despite this threshold, the data showed higher overall variance, likely due to underlying biological variability that remains undefined. Additionally, in Fig. 8A and B, we observed that as the ratio between the two analytes is closer to 1, the variance is significantly smaller, highlighting the importance of keeping that internal standard concentration close to the target analyte concentration. Lastly, these plots represented the high-precision measurements that were obtained when performing V × V quantification even after introducing biological variability in the experiment. Future work will focus on assessing the accuracy of V × V quantification as we identify a solution for quantitatively spraying the SIL standard.

## Conclusions

The feasibility of V × V quantification for glutathione in mouse liver was achievable by IR-MALDESI. This work demonstrated that voxel-based quantification has several promising advantages over the on-tissue calibration curves often employed for qMSI. This was particularly true in the sample preparation as well as when performing high specificity PRM-qMSI. Statistical analysis also indicated V × V quantification had high precision, but the accuracy of this method was limited by the quantitative deposition of the standard onto the glass slides. Overall, this qMSI method yielded promising results, and future work will focus on further optimizing and applying this method to model studies and pre-clinical applications.

## Data Availability

All data is available through NCSU’s Dryad system: https://doi.org/10.5061/dryad.b5mkkwhrk.
